# The upside of cumulative conceptual interference on exemplar-level mnemonic discrimination

**DOI:** 10.3758/s13421-024-01563-2

**Published:** 2024-05-06

**Authors:** Emma Delhaye, Giorgia D’Innocenzo, Ana Raposo, Moreno I. Coco

**Affiliations:** 1 CICPSI, Faculdade de Psicologia, Universidade de Lisboa, Lisboa, Portugal; 2 GIGA-CRC In-Vivo Imaging, University of Liège, Liège, Belgium; 3 Department of Psychology, Sapienza University of Rome, Rome, Italy; 4 IRCSS Santa Lucia, Roma, Italy

**Keywords:** Long-term visual memory, Semantic interference, Conceptual similarity, Visual similarity

## Abstract

Although long-term visual memory (LTVM) has a remarkable capacity, the fidelity of its episodic representations can be influenced by at least two intertwined interference mechanisms during the encoding of objects belonging to the same category: the *capacity* to hold similar episodic traces (e.g., different *birds*) and the conceptual *similarity* of the encoded traces (e.g., a *sparrow* shares more features with a *robin* than with a *penguin*). The precision of episodic traces can be tested by having participants discriminate lures (unseen objects) from targets (seen objects) representing different exemplars of the same concept (e.g., two visually similar *penguins*), which generates interference at retrieval that can be solved if efficient pattern separation happened during encoding. The present study examines the impact of within-category encoding interference on the fidelity of mnemonic object representations, by manipulating an index of cumulative conceptual interference that represents the concurrent impact of capacity and similarity. The precision of mnemonic discrimination was further assessed by measuring the impact of visual similarity between targets and lures in a recognition task. Our results show a significant decrement in the correct identification of targets for increasing interference. Correct rejections of lures were also negatively impacted by cumulative interference as well as by the visual similarity with the target. Most interestingly though, mnemonic discrimination for targets presented with a visually similar lure was more difficult when objects were encoded under lower, not higher, interference. These findings counter a simply additive impact of interference on the fidelity of object representations providing a finer-grained, multi-factorial, understanding of interference in LTVM.

**Supplementary Information:**

The online version contains supplementary material available at 10.3758/s13421-024-01563-2.

## Introduction

Seminal work on long-term visual memory (LTVM) has convincingly demonstrated that humans can store and successfully retrieve thousands of distinct pictures (Shepard, 1967; Standing, 1973; see also Brady et al., 2011, for a review) with great fidelity (Brady et al., 2008). Yet, the fidelity of these memory representations is influenced by different factors, with interference often suggested as a prominent mechanism inducing forgetting of the learnt information, due to similar memory representations overlapping in their content (for a review of the concept, see Craig et al., 2015; Dewar et al., 2007). In this context, when we are exposed to new information that is consistent with our expectations, i.e., similar to our pre-existing knowledge, its integration will lead to overlap and generalization of memory representations (Bein et al., 2023; Ritvo et al., 2019), with a consequent loss in their precision and specificity (Keresztes et al., 2018).

However, interference can be countered by hippocampal pattern separation (Yassa & Stark, 2011), which consists of building less confusable, more distinct memory representations by orthogonalizing overlapping information so making them more discriminable (Baker et al., 2016; Kesner, 2013; Motley & Kirwan, 2012; Wanjia et al., 2021). In the same line, differentiation, or repulsion, occurs when similar memory representations are co-activated during learning and are pushed away past the point of orthogonalization to boost their distinctiveness (Favila et al., 2016).

Interference, however, is a multi-dimensional construct, which has been extensively manipulated in the literature using a variety of experimental methods (see Son et al., 2021, for methodological innovations in this direction). Usually, the impact of interference on visual long-term memory representations is manipulated by either increasing the numerosity of exemplars from the same category that participants are exposed to (e.g., remembering four different animals vs. 16 different tools) or through the strength of their conceptual similarity (e.g., a dog and a cat are conceptually more similar than a dog and a bird despite being both animals). These two aspects of interference, which we describe in greater detail below, are two sides of the same coin. The current study precisely aims at conceptualizing both types of interferences under the same experimental approach and examine, at a finer granularity, their joint impact on the fidelity of memory representations.

### Capacity interference

As already touched on, a key experimental manipulation to increase interference is the progressive increase of the number of exemplars (e.g., pictures) from conceptually similar categories (e.g., a kitchen) concurrently stored in LTVM, which makes each exemplar compete for retrieval access. It is precisely the accumulation of conceptually overlapping episodes that blurs the encoding fidelity and so detrimentally impacts their successful retrieval. This idea dates to Bartlett (1932), but its more direct relation to interference comes from research by Konkle and collaborators (2010a, b), where participants studied varying numbers of pictures (objects and scenes) belonging to several semantic categories (e.g., one, four, 16 or 64 kitchen scenes) in preparation for a two-alternative forced-choice recognition test. The key observation was that an increase in the frequency of pictures within each semantic category resulted in a systematic decrement in recognition accuracy (see also Alvarez & Cavanagh, 2004, for effects of capacity load in short-term memory processes). Detrimental interference effects on the capacity to hold multiple memory traces have been replicated across different studies, using materials such as objects (e.g., Antonelli & Williams, 2017) or scenes (e.g., Melcher & Murphy, 2011), and with healthy as well as pathologically aged groups (Coco et al., 2021). In addition, there is evidence that increasing demands on the capacity for memorised items can be directly observed in eye-movement responses during encoding (Mikhailova et al., 2021) and event-related potentials during recognition (Poch et al., 2019).

### Conceptual similarity

Each semantic category is made of several possible exemplars, which may be more or less similar to each other in terms of their constituting conceptual features (e.g., a peacock and a robin are both birds even though they are conceptually distant). Still in the study by Konkle et al. (2010a), objects that were conceptually more distinctive within their category were less susceptible to the detrimental effect of interference on recognition accuracy, possibly because they shared fewer semantic features with other members of the same category. The confusability of items due to the overlap in their conceptual features is another factor that generates interference (see Hovhannisyan et al., 2021). The quantification of conceptual similarity (or distinctiveness) between objects can be obtained from feature norms (see Devereux et al., 2014, or McRae et al., 2005, for norms of words), which define object concepts by their constituent parts, or conceptual features (refer to Rosch, 1975, for seminal ideas about the featural organization of concepts). According to this approach, statistical regularities of the object features represented as frequency vectors can capture how they are represented and, consequently, their similarity computed as distances (e.g., cosine; Taylor et al., 2007, 2012). This measure can be used in memory research to quantify the conceptual similarity between old and new items presented at the test (Frick et al., 2023; Montefinese et al., 2015; Naspi et al., 2021).

### Pattern separation

The fidelity and the precision of memory representations can also be probed by evaluating the ability of participants to mnemonically discriminate two items that represent the same concept (e.g., two distinct frying pans) but that are visually similar (Kirwan & Stark, 2007; Leal & Yassa, 2018; Yassa & Stark, 2011). In this context, a highly specific memory of an encountered object (target) is necessary to correctly discriminate it from a similar but unseen new object (lure). When the visual similarity between the target and the similar lure is experimentally manipulated in these tasks, results consistently show a linear increase in false recognitions with the increase in target-lure similarity (Anderson et al., 2016; Motley & Kirwan, 2012). Among the proposed explanations for this finding is an excessive reliance on gist, i.e., semantic memory, at test, in the absence of precise perceptual mnemonic representations (Koutstaal & Schacter, 1997), or a failure of pattern separation leading to a pattern completion mechanism, that is, the use of a partial or degraded cue to retrieve a previously stored memory (Anderson et al., 2016).

### Memory fidelity in the face of interference

Although several studies have manipulated visual similarity between target and lure to examine how this type of interference influences successful retrieval (e.g., Anderson et al., 2016; Motley & Kirwan, 2012), to our knowledge, only a few studies tested the interaction between the three types of interferences we just described (i.e., capacity, conceptual and visual). In the context of memory for objects, Naspi et al. (2021), for example, showed that conceptually more similar objects were less likely remembered, and their associated lures were more likely rejected but only when visually distinct from the target, but this study did not examine the effect of capacity interference. Poch et al. (2019), instead, showed that capacity interference significantly hinders mnemonic discrimination between targets and similar lures, but did not manipulate either the conceptual similarity or the visual similarity between these two visual objects.

In sum, our literature review points to two key manipulations inducing interference at encoding that act upon the fidelity of memory representations: the capacity to hold multiple episodic traces belonging to the same semantic category and the conceptual similarity between these traces. Moreover, a third type of interference can be generated during retrieval by manipulating the visual similarity between two conceptually identical objects (one stored in memory and the other unseen) in pattern separation tasks. These three types of manipulations have, to the best of our knowledge, been independently examined. On the one hand, the similarity between feature norms has been used to operationalise conceptual interference during encoding but without directly considering the effect arising when the capacity also varies (e.g., Naspi et al., 2021). On the other hand, capacity was varied systematically also during encoding but there was no direct control on the conceptual similarity between the exemplars of the same category (Antonelli & Williams, 2017; Coco et al., 2021; Konkle et al., 2010a; Mikhailova et al., 2021; Poch et al., 2019). Here, we suggest that these two types of interferences, generated during the encoding of the visual objects, inevitably co-vary. Increasing the number of exemplars of the same semantic category (i.e., set size) should indeed necessarily also lead to an increase in conceptual interference, and so naturally add to the effect brought by the conceptual similarity among exemplars stored in LTVM. Our study precisely attempts to investigate the cumulative interference induced by the interplay of these factors, which builds up during the encoding of a stream of visual objects. We assessed the fidelity of the memory representations for seen targets by using lures representing the same concept but choosing a different visual exemplar, thereby generating retrieval interference. This manipulation allowed us to tap into the mechanisms of pattern separation and so helped us probe the precision of mnemonic object representations. The visual similarity between targets and lures was also computed using an algorithmic approach (Huebner & Gegenfurtner, 2012; Neumann & Gegenfurtner, 2006; Zelinsky, 2003), and we introduced this measure as a predictor in our analysis to examine its interplay with conceptual interference.

### Current study

**Fig. 1:**
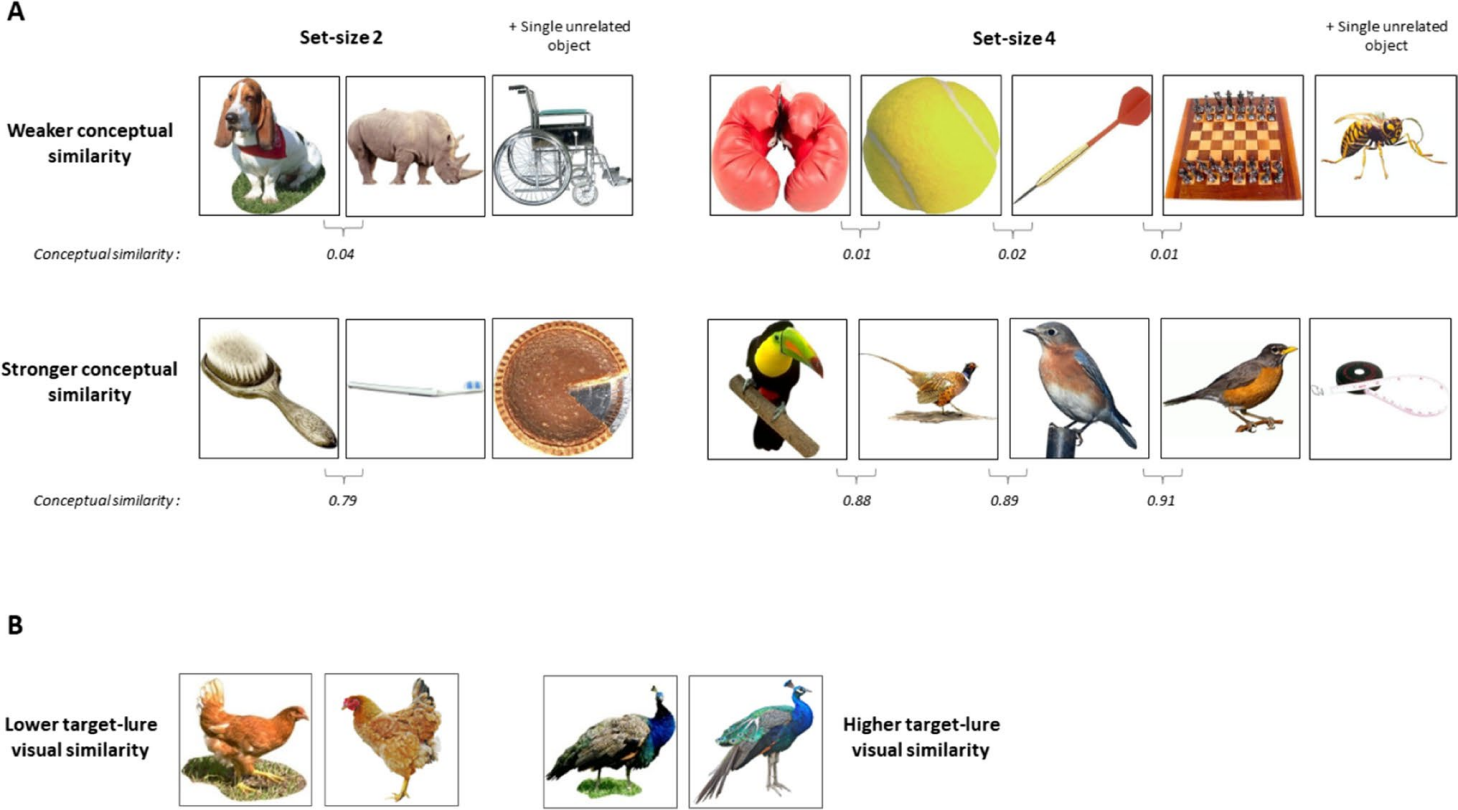
**A** Visual examples of capacity sets (set-size 2 left; set-size 4 right), and conceptual similarity between consecutive items within a set (weaker similarity, top; stronger similarity, bottom) during encoding. Each set was followed by an unrelated single object. (**B**) Visual examples of visually similar and dissimilar lures during recognition

The main methodological innovation of our old/new recognition memory task is that we concurrently manipulated the number of object exemplars from the same semantic category administered during encoding alongside their conceptual similarity as measured by their similarity in terms of shared features (Hovhannisyan et al., 2021). First, the sequence of objects presented during the encoding phase was administered such that they belonged to one of two possible levels of capacity (sets of two vs. four objects). Second, even if objects always belonged to the same category (e.g., a set of different *electrical appliances*), the degree of conceptual overlap within each capacity set was systematically quantified (e.g., a *washing machine* shares more features with a *dryer* than with a *toaster*). Third, the degree of conceptual similarity between pairs of objects presented sequentially increased within each capacity set, to accumulate greater levels of interference. Consequently, as we assume that capacity load and conceptual similarity might jointly build up interference, we conceived an innovative single measure that accounts for the *cumulative conceptual interference*, within each set, during encoding (see *Statistical analysis* section for details about this measure and the framework of analysis). To break the build-up of interference during encoding between consecutive sequences of conceptually related objects, we borrow the concept of *interference release*, which posits that a change in semantic content within a study list to be held in working memory will produce a release of interference build-up (Wickens, 1970). More precisely, we introduced a categorically and conceptually unrelated single object between each capacity set (see Fig. 1 for a visualisation of the design), thus releasing the interference building up through the stream of related objects.

Our main expectation is that higher cumulative conceptual interference will result in lower recognition accuracy since strong interference would imply a decrease in recognition hit rate (e.g., Konkle, et al., 2010a). Also, high visual similarity between target and lure should itself generate interference, leading to a higher false alarm rate (e.g., Motley & Kirwan, 2012), especially following encoding under high cumulative conceptual interference, indicating possibly less effective pattern separation during encoding.

## Methods

### Participants

Seventy-six participants, native Portuguese speakers, took part in the study in exchange for course credits. The sample size was derived from previous literature implementing similar manipulations and designed such that our study would be approximately three times larger in terms of observations gathered during recognition (N = 30,447 vs. N = 10,800 in Naspi et al., 2021). Data from four participants were excluded due to below-chance performance either during encoding (n = 1) or during memory recognition (n = 3). The data of one further participant was excluded because of a machine error (i.e., a corrupted log file). Analyses were thus performed on the remaining 71 participants (mean age = 20.32, *SD* = 5.78, range =18–47 years; 61 females). The study was approved by the local Ethics Committee before commencing data collection.

### Material

From the real object pictures database by Hovhannisyan et al. (2021), we selected 987 targets of 27 different categories and used the feature norms available therein to operationalise our experimental variable of conceptual similarity. In this database, each object is represented as a vector of conceptual features (e.g., “is made of metal”) and a matrix of all objects’ similarities was computed by taking pairwise cosine distances.

From this pairwise similarity matrix, we selected sets of four objects and their conceptual similarity measures within each category. Furthermore, objects were organised in sequential order within each set such that the object that followed always had a stronger interference with the one presented just before.^1^ Then, sets of two were created by dividing the sets of four into two sets of two and were counterbalanced such that each object was never presented more than once to the same participant. The remaining objects were used as single unrelated objects to “break” the interference and avoid participants developing an automatic response strategy during the incidental encoding phase. So, an unrelated object was paired with a given set such that it was from a different semantic category and had a very low conceptual similarity with the object that preceded and followed it (see Fig. 1 for a visualization). Moreover, we made sure that the capacity set that preceded and followed the single unrelated objects was always of a different semantic category. An additional 987 different exemplars matching the same object concepts were selected as lures from other available databases (Adlington et al., 2009; Brady et al., 2008; Brodeur et al., 2014; Konkle & Oliva, 2012; Kovalenko et al., 2012; Moreno-Martínez & Montoro, 2012). The visual similarity between the target and its matched lure was computed a posteriori using an algorithmic approach, i.e., the bank of local analyzer responses (Zelinsky, 2003; see also Cimminella et al., 2020, for an example in the context of a visual search task).

### Procedure

**Fig. 2:**
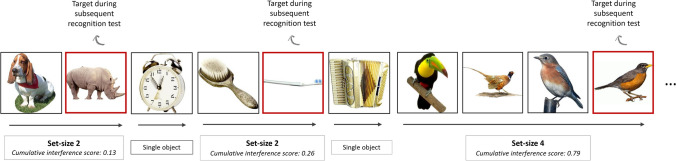
Illustration of the encoding phase, composed of sets of two and four related items with increasing levels of conceptual similarity within each stream. Each set is presented as an uninterrupted stream of standalone objects interleaved by an unrelated object. For each stream, we calculated an index of cumulative interference (see the *Statistical analysis* section part for details on the calculation)

In the encoding phase, participants were presented with a total of 880 object pictures belonging to either a set of four (440 objects across 110 sets), a set of two (220 objects across 110 sets) or single objects (220 objects, one between each set of two or four). Sets (two and four) were displayed as uninterrupted streams of object pictures, in a fixed order with increasing similarity within each set, and the last object of each set was followed by an unrelated, single, “break” object. See Fig. 2 for an illustration of the sequence. Participants were instructed to determine, for each object, whether it was natural or manufactured, and were not informed about the subsequent memory task so that encoding was incidental. This design choice is in line with previous research supporting that incidental learning during encoding gives rise to levels of subsequent memory performance that are comparable to intentional learning (e.g., Craik, 2023), even in the face of interference (Oberauer & Greve, 2022; but see Dames & Popov, 2023, for arguments in favour of a boost in memory following intentional learning). Each encoding trial started with a 500-ms fixation cross followed by an object being displayed for 1 s, and then a question screen during which participants pressed one of two keys (the “a” and the “l”) to provide their response. Keys were counterbalanced across participants. If the answer was not provided within 3 s, the screen disappeared, the next trial started, and a null response was logged. After the encoding phase, there was an intermediate phase during which participants completed two short-term memory tasks: a forward digit-span task (mean = 5.78, *SD* = 1.59) and a Corsi block tapping task (mean = 6.34, *SD* =1.56). This phase was included so that participants remained engaged during the retention break while preventing them from rehearsing, and so that the distracting tasks would not interfere with the materials from the experimental task^2^.

Participants were then informed about the recognition phase and presented with the last object of each encoded set (i.e., 110 objects encoded in streams of four and 110 objects encoded in streams of two) as well as 55 single break objects, randomly selected. As mentioned in the *Material* section, each object presented in the encoding phase (i.e., old) was matched with a different exemplar (i.e., new) of the same object (e.g., another washing machine). Thus, participants responded to 550 objects during the recognition phase, half targets (275) and half lures (275). Participants had a maximum of 4 s to provide their old or new response, otherwise, a null response was logged, and the next recognition trial was shown. Following each old/new recognition response, they were additionally asked to provide a confidence judgement on a 2-point scale^3^ (sure/unsure). All items were distributed following a Latin-square rotation to ensure that all objects across all conditions were seen at the recognition phase, resulting in a total of 24 randomization lists.

### Statistical analyses

Our main analysis focuses on recognition responses and was carried out separately for seen (old) objects and unseen (new) objects (but see Online Supplementary Material (OSM) C for analyses on d prime and criterion). In the terminology of signal-detection theory, correct responses to seen objects correspond to *hits*, while those to unseen objects to *correct rejections*. Both measures were used as dependent measures in our analysis. As predictors, we considered *cumulative conceptual interference* (described hereafter) and *visual similarity* (only for a subset of analyses, see below) both represented as continuous variables. To gauge potentially incremental effects of interference as a function of the interplay between conceptual similarity and capacity on memory processes, we derived it as a cumulative measure based on the association values of objects as sequentially presented in the encoding stream within their category capacity (i.e., two and four set-sizes). For example, suppose a participant viewed sequentially a set of four objects (e.g., *microwave*, *dishwasher*, *dryer* and *washing machine*) from the same category (e.g., *appliances*) before the single object (e.g., *apple*) from an unrelated category (i.e., *fruit*) broke the interference build-up. We summed the cosine distance between vectors of conceptual features for contiguously presented objects (i.e., their similarity) to obtain the cumulative value of interference on the target object (i.e., the last object of the set). So, in the example above, we have the following similarity scores: microwave-dishwasher (.55), dishwasher-dryer (.69), dryer-washing-machine (.69); the sum of this value would be 1.93, which would correspond to the amount of cumulative interference generated on the target object dryer (i.e., the seen object to be recognized). So, a greater number of more conceptually similar objects would lead to greater cumulative interference. These cumulative sums were then normalised to range between 0 and 1 by dividing all scores to the maximum observed across the entire dataset.^4^

As the target and the lure objects were two visual exemplars of the same, conceptually identical, object (e.g., two different-looking frying pans), the correct rejection of a lure could be influenced by their visual similarity to the target.^5^ So, visual similarity between the target and lure object was computed using the bank of local analyser responses (Zelinsky, 2003), which provides an aggregate score ranging between 0 and 1 measured based on low-level visual features of the objects (colour, orientation and size). Moreover, during the recognition phase, the target and matched lure object could equally appear either as a first or as a second object in the randomization list. Thus, when the target object was presented *after* the lure object during the recognition phase (i.e., second object), its successful recognition (i.e., hit) could have been hindered by the visual similarity with the previously seen lure (first object). So, we took a subset of the data where the lure was presented first (N = 7,381) and examined how its visual similarity to the target would have impacted target recognition (i.e., hits).

Generalised linear-mixed regressions (GLMER) were used as the analytical framework to gather inferences from our data using a binomial link function given our dependent variable (i.e., hits and correct rejections). We implemented the GLMER on the lme4 package (Bates et al., 2015) in R (version 4.2.2). The predictors considered in the model were: *Cumulative Interference* (continuous variable from 0 to 1) and when the dependent measure was either correct rejections or hits for the subset of data where the lure was presented before the target during the recognition phase, we included *Visual Similarity* as predictor, indicating the low-level perceptual overlap between the seen and unseen object (continuous variable from 0 to 1). The random variables included in the models were Participants (71), Semantic Category^6^ (27) and Recognition Order^7^ (2).

We first built models that had a maximal fixed and random effect structure, i.e., all fixed effects are introduced as main effects and in interaction, and we estimated the variance of our random effects both as intercept, and as uncorrelated slopes of our predictors (see Barr et al., 2013). Then, we used backward model selection using the step function from the lmerTest package (Kuznetsova et al., 2017) to obtain the final model that is most parsimonious in its structure (i.e., all models converged) while best fitting our data (see Matuschek et al., 2017, for advocating this approach). Of these models, we also compute standardised β coefficients, which can be used to compare the relative proxies to effect size, the relative strength of each predictor, as well as the confidence intervals to judge the uncertainty in our estimates (refer to Luke, 2017, for an interesting analysis). Tables 1 and 2 report the full model specification using the Wilkson notation including the coefficients, standardized coefficients, confidence intervals, standard errors, *z*-values and *p*-values based on asymptotic Wald tests computed using the lmerTest package.

**Table 1:** Generalized linear mixed-effects model for Hits of all target objects, and also focusing on the target objects that were presented in the recognition phase after the lures as predicted by Cumulative Interference (continuous, from to 0 to 1) and Visual Similarity (continuous, from 0 to 1 and introduced in the model only for targets presented after lures). The random variables introduced as intercept and slopes were Participant (71), Semantic Category (27) and Recognition Order (1, 2)

Dependent variables	Predictor	β	Std. β	CI (2.5%; 97.5%)	SE	z-value
Hits	Intercept	0.77	0	0.45; 1.09	0.16	4.78***
	Cumulative Interference	-0.36	-0.21	-0.63; -0.1	0.13	-2.68**
Hits (only target objects presented after the lure)	Intercept	0.34	0	-0.09 ; 0.77	0.22	1.54
	Cumulative Interference	0.49	0.28	-0.24 ; 1.22	0.37	1.31
	Visual Similarity	0.67	0.23	0.05; 1.3	0.31	2.12*
	Cumulative Interference × Visual Similarity	-1.53	-0.54	-2.74; -0.32	0.61	-2.49*

The final model formulas in Wilkson notation, resulting from stepwise backward selection are:

a) Hits ~ Cumulative Interference + (0 + Cumulative Interference | Semantic Category) + (1 | Semantic Category) + (1 | Recognition Order) + (1 | Participant)

b) Hits (targets presented after lures) ~ Cumulative Interference + Visual Similarity + (1 | Participant) + (1 | Semantic Category) + (0 + Cumulative Interference | Semantic Category) + (0 + Visual Similarity | Semantic Category) + (0 + Visual Similarity| Participant)

*** p < .001, ** p < .01 * p < .05

**Table 2:** Generalized linear mixed-effects model for Correct Rejections (unseen objects) as predicted by Cumulative Interference (continuous, from to 0 to 1) and Visual Similarity (continuous, from 0 to 1 and introduced in the model only for unseen objects). The random variables introduced as intercept and slopes were Participant (71), Semantic Category (27) and Recognition Order (1, 2)

Dependent variable	Predictor	β	Std. β	CI (2.5%; 97.5%)	SE	z-value
Correct rejections	Intercept	2.18	0	1.71; 2.64	0.23	9.2***
	Cumulative Interference	-1.39	-0.88	-2.02; -0.77	0.31	-4.4***
	Visual Similarity	-2.1	-0.79	-2.74; -1.39	0.34	-6***
	Cumulative Interference x Visual Similarity	2.71	1.03	1.74; 3.67	0.49	5.5***

The final model formula in Wilkson notation, resulting from stepwise backward selection is:

‘Correct rejections ~ Cumulative Interference + Visual Similarity + Cumulative Interference x Visual Similarity + (0 + Cumulative Interference | Semantic Category) + (0 + Visual Similarity | Semantic Category) + (1 | Semantic Category) + (1 | Recognition Order) + (1 | Participant) ’

*** p < .001, ** p < .01 * p < .05

The script and data to replicate the results presented here are available on the Open Science Framework (https://osf.io/5rh2e/).

## Results

**Fig. 3:**
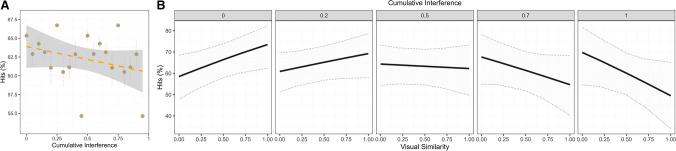
**A** Percentage hits (i.e., correctly recognizing a seen object) as a function of Cumulative Interference. The points are aggregated values across participants and items observed within ten equally spaced bins of interference scores (i.e., 0–0.1, 0.1–0.2, 0.2–0.3, etc.). The error bars reflect the standard error around the mean. The dashed line instead represents the linear fit while the shaded bands reflect its 95% confidence intervals (upper, lower). (**B**) Percentage hits as a function of Visual Similarity across levels of Cumulative Interference, organized as bins, each representing intervals of increasing interference (i.e., 0–0.2; 0.2–0.5; 0.5–0.7, 0.7–0.8; 0.8–1) obtained from the underlying quantile distribution (i.e., 0%, 20%, 40%, 60%, 80%, 100%). The solid line represents the linear fit while the dotted lines its 95% confidence intervals (upper, lower)

On the hits, we observed a significant main effect of Cumulative Interference, where seen objects encoded under greater interference were less likely to be correctly recognized (see Fig. 3A for visualisation and Table 1 for the modelling output). When isolating hits for target objects appearing after the lures in the recognition phase, and so including Visual Similarity in the analysis, the recognition of the target objects significantly improved as the similarity with the lure objects increased. This main effect was, however, modulated by a significant interaction with Cumulative Interference, whereby the stronger the conceptual interference, the worse the recognition of the target object for increasing visual similarity with the lure (see Table 1 for model coefficient and Fig. 3B for a visualisation of the effect). This finding indicates that visual similarity promotes, rather than hinders, the identification of the target when presented after the lure. However, such an effect is substantially reduced by the cumulative interference, which confirms it to be detrimental to the recognition of the target held in LTVM.

**Fig. 4:**
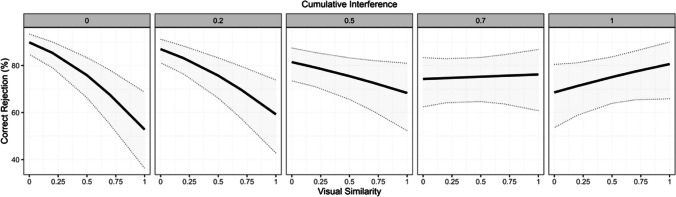
Percentage correct rejections (i.e., correctly rejecting an object as unseen) as a function of Visual Similarity across levels of Cumulative Interference, organized as bins, each representing intervals of increasing interference (i.e., 0–0.2; 0.2–0.5; 0.5–0.7, 0.7–0.8; 0.8–1) obtained from the underlying quantile distribution (i.e., 0%, 20%, 40%, 60%, 80%, 100%). The solid line represents the linear fit while the dotted lines its 95% confidence intervals (upper, lower)

On the correct rejections, we found significant main effects of Cumulative Interference, whereby unseen objects were more likely incorrectly judged as seen when the seen object was encoded under greater interference; and of Visual Similarity, whereby unseen objects were more likely incorrectly judged as seen when highly similar to the seen objects. Most interestingly, there was a significant interaction between Cumulative Interference and Visual Similarity. As can be seen from Fig. 4, difficulties in mnemonic discrimination leading to incorrectly judging an unseen, visually similar, object as seen, were particularly strong when cumulative interference was low, while the detrimental effect of visual similarity became weaker for increasing levels of interference (refer to Table 2 for the modelling output).

Finally, in two control analyses, first, we ensured participants did not develop an automatic response strategy during the incidental encoding by showing that categorization accuracy for break objects (mean accuracy ≈ 95%) was not influenced by the preceding Set Size (β = -0.06, z-value = -0.3, p-value = 0.7). Finally, we showed that the build-up of semantic interference along the entire session, i.e., the frequency of exemplars belonging to the same semantic category, did not interact with the effects of Cumulative conceptual Interference (see OSM B for details and results).

## Discussion

Humans have remarkable long-term memory for visual information (e.g., Shepard, 1967; Standing, 1973); however, different episodic representations of similar events (e.g., remembering which fruit is in the fridge) may overlap in their content, thus interfering with their efficient pattern separation, and so hinder the subsequent successful retrieval of each episodic instance (e.g., there is an apple and not a pear in the fridge). The existing literature suggests that there are at least two intertwined types of informational overlap that could create interference while encoding visual information in memory: capacity (i.e., the number of exemplars from the same category) and conceptual similarity (i.e., how many features two concepts share). Moreover, each object has a unique perceptual appearance (e.g., a green vs. a red apple) and retrieval processes are known to be hindered by interference that may arise when target objects must be discriminated from lures merely based on their visual similarity.

Most previous research has independently investigated the impact of these different types of interferences on the fidelity of episodic memory representations and the discrimination mechanisms operating to successfully recall them. In the current study, instead, we jointly manipulated the interference generated by capacity and conceptual similarity during encoding, which is a methodological innovation compared to previous research, and tested whether the interference arising from the accumulation of these two factors may impact the fidelity of memory representations. We did so by administering sequences of pictures of real objects to be incidentally memorized that were grouped within the same semantic categories but into different capacity sets (i.e., two or four) and whose conceptual similarity was quantified using measures of featural similarity. We derived an integrated measure of cumulative interference from these two factors to gauge the potentially incremental effects of interference on the encoding fidelity of their mnemonic representations. Additionally, we tapped into discrimination mechanisms operating during memory retrieval to assess the fidelity of memory representations by administering target-lure pairs which always represented the same concept but varied in their visual appearance (e.g., two different fridges).

In line with previous findings (e.g., Konkle et al. 2010a; Mikhailova et al., 2021), but using a novel continuous metric integrating capacity and conceptual interference, we found that under greater cumulative conceptual interference, recognition of seen objects (i.e., hits) significantly decreases. It is important to stress that this effect of cumulative conceptual interference on the hits is independent of a more global category interference effect building up across the entire experimental session (see OSM B). As recognition was assessed with a yes/no paradigm, we computed correct rejection rates and demonstrated that they also decrease as cumulative inference increases. Importantly, these effects cannot be interpreted as shallower encoding of objects presented within larger sets, as encoding categorization accuracy of the break objects was not influenced by the numerosity of the preceding set size. However, our study also brings an important nuance to this observation by examining how, and whether, cumulative conceptual interference at encoding interacts with the retrieval interference generated by the visual similarity between targets and lures.

Indeed, we replicated the finding that greater visual similarity between targets and lures decreases the correct rejection of lures (Anderson et al., 2016; Motley & Kirwan, 2012; Naspi et al., 2021), but this effect is modulated by the cumulative interference generated at encoding. Critically, the high visual similarity of the lure with the target indeed hindered its overall correct rejection, but this effect was particularly strong when targets were encoded under low cumulative interference, while it nearly disappeared, if not reversed, when targets were encoded under conditions of high conceptual interference. Moreover, the pattern is exactly the opposite on the hits when the lure is presented before the target: in that case, targets encoded under lower cumulative interference are better recognized when lures preceding them are highly similar in visual appearance. In other words, altogether, participants got more liberal under lower encoding interference, when retrieval interference was higher. This effect is, however, substantially reduced by cumulative conceptual interference, which confirms its detrimental impact on the fidelity of the representations for object exemplars stored in LTVM. Importantly, these effects cannot be simply ruled out by the difficulty of the task, as, even under the greatest encoding interference and the highest visual similarity between target and lure, participants performed well above chance. Thus, the presence of interference generated by encoding targets into conceptual sets that were strongly related promoted more detailed, finer-grained, exemplar-level representations that protected against detrimental effects of target-lure visual similarity, and a change in response strategy towards being more conservative when looking at correct rejections of unseen objects. This supports previous evidence that conceptual relationships between memorized objects may be, to a certain degree, beneficial to their encoding in visual working memory (O’Donnell et al., 2018), and this positive boost carries over to their long-term episodic memory representations (Greve et al., 2019).

A different angle for interpreting these results is to consider the distinctions between memory integration and pattern separation. When new information (e.g., a visual object) needs to be integrated into our pre-existing knowledge structure (i.e., here, the sequential activation of conceptually similar objects), it will lead to the generalization and consequently to a loss of memory specificity for that particular new episodic exemplar. On the opposite side, pattern separation reinforces memory specificity and precision for new information, while repulsion effects push representations for similar exemplars away from each other, thus deforming the precision of each instance while ensuring their distinctiveness (Bein et al., 2023; Keresztes et al., 2018; Ritvo et al., 2019). So, it is conceivable that the interplay between memory integration and pattern separation is modulated by the extent to which incoming information matches prior knowledge. Pattern separation would occur mainly in the face of higher encoding interference, while memory integration would instead be more likely to occur at lower levels of encoding interference.

The absence of visual similarity effects on mnemonic discrimination for high levels of conceptual interference is also in agreement with Konkle et al. (2010a), who did not show any effect in this direction. Yet, the major novelty of our findings is that interference does not simply display a cumulative and additive effect due to conceptual or capacity overlap, but there are benefits to forming memory representations in conceptually related environments. Another interpretation could be that increased conceptual interference recruits more cognitive resources, which may attenuate the effect of visual similarity.

Even if our analyses of correct rejections revealed an interesting pattern, the use of old/new paradigms to investigate memory processes has recently come into question (Brady et al., 2022). To consolidate the findings of the present study, future research with the same methodological approach but testing memory recognition in two alternative forced-choice as used in previous studies manipulating interference (e.g., Konkle et al., 2010a; Poch et al., 2019) is needed. Another open question from our study is the role played by incidental learning and whether our findings can be replicated when participants are actively and explicitly asked to memorize the stream of visual objects (Dames & Popov, 2023; Oberauer & Greve, 2022). Our experimental design was also challenged by the necessity to manipulate the conceptual similarity of hundreds of different objects while balancing their numerosity within each semantic category. Although we demonstrated that cumulative conceptual interference goes above and beyond interference building globally for each semantic category across the experimental session, we would welcome research that manages to better equalize this aspect in their study design. Another core question the study also leaves is about the neural correlates of this new index of cumulative interference, and how it relates to hippocampal pattern separation (Kesner, 2013). As the P300 is known to be involved in recognition processes (Friedman et al., 2001), and characteristics of this component (e.g.., latency) point at the strength of recognition (Johnson et al., 1985), we may expect its amplitude to reduce, or its latency to delay, for increasing cumulative conceptual interference. Finally, our investigation contemplated LTVM for stand-alone single objects, but it is known that the visual context can play a key role in mediating their encoding and access (e.g., Evans & Wolfe, 2022 and see Castelhano & Krzyś, 2020 for an interesting review). Thus, more work is needed to clarify how the cumulative interference proposed in this study would play on recognition memory for objects embedded into richer and more structured visual contexts.

In sum, our study proposed a new theoretical and methodological approach to tackle interference in the long-term memory of visual objects. Results replicated the classical effect of interference on decreasing hits as well as correct rejections. Most importantly though, we demonstrated that an additive mechanism of interference is not sufficient to explain our findings, as high levels of conceptual interference at encoding were associated with a reduced detrimental effect of visual similarity of the lures on memory performance, which suggests that high cumulative interference at encoding may promote a finer-grained exemplar-level encoding. Taken together, our findings depict a finer-grained, multi-factorial understanding of interference mechanisms in LTVM.

## Supplementary Information

Below is the link to the electronic supplementary material.

Supplementary file1 (DOCX 418 KB)

## Footnotes

1 Note, this was the case for all items in the strong conceptual similarity trials. For the weak conceptual similarity trials, this was more challenging to implement as values of conceptual similarity approached 0. Nevertheless, on average, conceptual similarity values were as follows: similarity between first and second items, mean _high_ = 0.59 (*SD* = 0.20), mean _low_ = 0.06 (*SD* = 0.11), *t*(218) = 23.98, *p* < .001; between second and third items, mean _high_ = 0.65 (*SD* = 0.17), mean _low_ = 0.18 (*SD* = 0.17), *t*(218) = 19.60, *p* < .001; between third and fourth items, mean _high_ = 0.77 (*SD*=0.11), mean _low_ = 0.25 (*SD* = 0.22), *t*(218) = 22.04, *p* < .001.

2 The relationship between the intermediate phase and the main experimental task is not the focus of the current paper, and so the results from this phase will not be further discussed.

3 Confidence responses significantly correlate with recognition responses, even if the strength of association is weak [r(30,445) = .13, p < .01]. When analysed as the dependent measure to predict cumulative conceptual interference using GLMER, we obtain the same trend observed on recognition responses but it is only marginally significant (β = -.11, p = 0.07). For these reasons, we decided not to provide a full report of this dependent measure in the article.

4 See Online Supplementary Material (OSM) D for an alternative computation of the Cumulative Interference (resulting in equivalent results).

5 Note, visual similarity of seen targets with themselves will obviously lead to a value of 1.

6 We opted to use Semantic Category rather than Individual Objects as random variable because in this latter case, we have much fewer data points, which often led to models that did not converge, especially when introducing random slopes (e.g., 0 + Cumulative Interference | Individual Object).

7 As seen and unseen objects are visually different exemplars of the same object (i.e., two different dishwashers), we explicitly control whether responding to its first or second presentation impacts its recognition by including this factor as random intercept in our models.
