# Supplementary material for: The upside of cumulative conceptual interference on exemplar-level mnemonic discrimination
Source: Mem Cognit. 2024 May 6;52(7):1567–78. doi: 10.3758/s13421-024-01563-2 (PMC11522113; doi:10.3758/s13421-024-01563-2)
Supplement: Supplementary file 1 — Supplementary file1 (DOCX 418 KB) [file 13421_2024_1563_MOESM1_ESM.docx]

**Supplementary Materials**

for

**The upside of cumulative conceptual interference on exemplar-level mnemonic discrimination**

^1^ Emma Delhaye, ^2^ Giorgia D’Innocenzo, ^2^Ana Raposo, ^3,4^ Moreno I. Coco

^1^ GIGA-CRC In-Vivo Imaging, University of Liège, Belgium

^2^ CICPSI, Faculdade de Psicologia, Universidade de Lisboa, Portugal

^3^ Department of Psychology, Sapienza University of Rome, Italy

^4^ IRCSS Santa Lucia, Italy

Correspondence can be sent to Dr Emma Delhaye (emma.delhaye@uliege.be) or Dr Moreno I. Coco ([moreno.cocoi@gmail.com](mailto:moreno.cocoi@gmail.com))

**Supplementary Material A**

*Analyses of hits and correct rejections based on the experimental design variables.*

In this section, we replicate the analyses of hits and correct rejections presented in the main text but instead of aggregating our experimental variables into a unique continuous index summarizing the cumulative conceptual interference participants were subjected to, we follow the explicit factorial design implemented in our study. As for the main text, we use generalized linear effect models and have as dependent measures, *hits* (a binomial indicating whether a seen, old, object was correctly recognized) and *correct rejection* *(*a binomial indicating whether an unseen, new, object was correctly rejected). The predictors included in the model, as fixed effects, are *Set Size* (2 and 4, with 2 as the reference level), *Conceptual Relatedness* (weak and strong, with weak as the reference level); and only for the analysis of the correct rejection also *Visual Similarity* (low vs. high, determined the levels based on a median split of the continuous measure and setting low as the reference level). The random effects considered are Participant (71), Semantic Category (27) and Recognition Order (1, 2) evaluated as intercepts and slopes. Models are initially built with a maximal random effect structure and fixed effects introduced as mains and in all possible interactions (Barr et al., 2013). Then, the models are backwards selected using the step function in lmerTest (Kuznetsova et al., 2017) to achieve the most parsimonious model explaining our data (Matuschek et al., 2017). Post-hoc tests, Tukey adjusted, to assess the interaction terms are obtained using the pairs function from the emmeans package (Searle et al., 1980 for a description of the original method) and report only the most relevant significant comparisons.

On hits, we observed significantly better recognition performances for target objects encoded in set sizes of 2, compared to 4, especially when such objects were encoded into strongly conceptually related sets compared to weakly related sets (Weakly related – Set Size 2 vs. Strongly related – Set Size 4, β = 0.33, z ratio = 3.3, p < 0.001).

On correct rejections, we also confirm that lures more visually to the target objects are falsely alarmed more as seen objects than visually dissimilar objects. This effect is compensated by conceptual similarity and set size, whereby, correct rejections increase for visually similar lures when targets are encoded into larger sets of conceptually related objects (Set Size 2 – Low Visual Similarity vs. Set Size 4 – High Visual Similarity, β = -0.37, z ratio = -3.68, p < 0.001).

These results fully corroborate what was presented in the main text where Set Size and Conceptual Relatedness were integrated into a unique continuous predictor (refer to Figure 1S and Table 1S below, for visualization and inferential statistics).

**Figure 1S**

Boxplot of percentage for the hits (seen objects, panel A) and correct rejections (unseen objects, panel B) as a function of the Set Size (2, 4) split by Conceptual Relatedness (*weak*, grey colour; *strong*, red colour) compared within each panel. Only for correct rejection (panel B), we further distinguish the Visual Similarity of the lure from the target (low and high) as categorically defined using a median split of its continuous score. The hinges of the boxes represent the 25th and 75th percentiles of the measure (lower and upper quartiles). The horizontal line represents instead the median of the distribution. Each dot indicates the by-participant average for that factor.


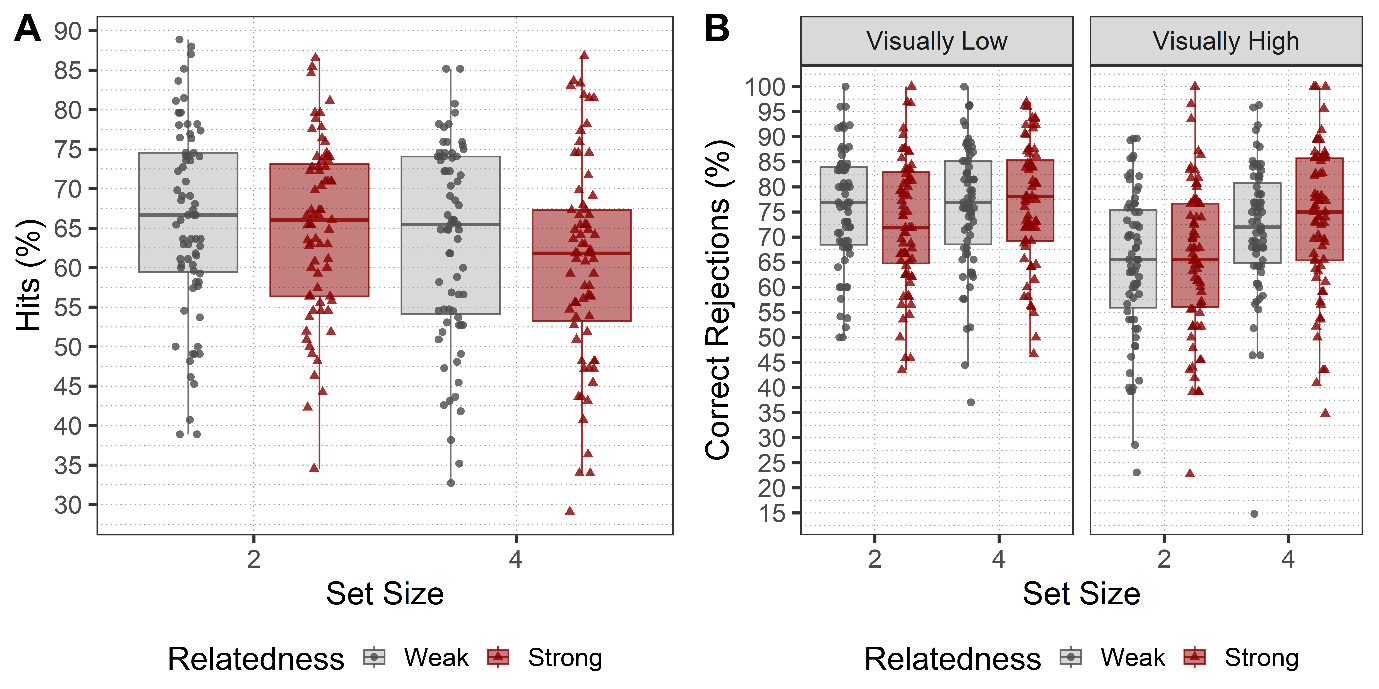


**Table 1S**

Generalized Linear Mixed-effects Model for Hits (seen objects) and Correct Rejections (unseen objects) as predicted by Set Size (2, 4, with 2 as the reference level), Conceptual Relatedness (weak, strong, with weak as the reference level) and Visual Similarity (low, high, with low as the reference level). The random variables introduced as intercept and slopes were Participant (71), Semantic Category (27) and Recognition Order (1, 2).

| Dependent Variable | Predictor | β | Std. β | CI  (2.5%; 97.5%) | SE | z-value |
| --- | --- | --- | --- | --- | --- | --- |
| Hits | Intercept | 0.86 | 0 | 0.53; 1.19 | 0.16 | 5.16*** |
|  | Conceptual Relatedness (strong) | -0.26 | -0.27 | -0.42; -0.1 | 0.08 | -3.19** |
|  | Set Size (4) | -0.23 | -0.24 | -0.39; -0.07 | 0.08 | -2.83** |
|  | Conceptual Relatedness (strong) x Set Size (4) | 0.15 | 0.07 | 0; 0.3 | 0.07 | 2* |
| Correct rejections | Intercept | 1.29 | 0 | 0.28; 0.9 | 0.17 | 7.44 |
|  | Conceptual Relatedness (strong) | -0.08 | -0.09 | 0.04; 0.44 | 0.1 | -0.75 |
|  | Set Size (4) | -0.02 | -0.03 | 0.17; 0.57 | 0.1 | -0.23 |
|  | Visual Similarity (high) | -0.69 | -0.78 | -1.04; -0.34 | 0.17 | -3.91 |
|  | Conceptual Relatedness (strong) x Visual Similarity (high) | 0.32 | 0.31 | 0.13; 0.51 | 0.09 | 3.34 |
|  | Set Size (4) x Visual Similarity (high) | 0.39 | 0.38 | 0.21; 0.58 | 0.09 | 4.25 |

*Notes*: The final model formulas in Wilkson notation, resulting from stepwise backward selection are:

a) Hits ~ Conceptual Relatedness + Set Size + (0 + Conceptual Relatedness | Semantic Category) + (0 + Set Size | Semantic Category) + (1 | Recognition Order) + (1 | Participant)

b) Correct rejections ~ Conceptual Relatedness + Set Size + Visual Similarity + Visual Similarity x Conceptual Relatedness + Visual Similarity x Set Size + (0 + Conceptual Relatedness | Semantic Category) + (0 + Set Size | Semantic Category) + (1 | Recognition Order) + (1 | Participant)

(*) p < .10, *p < 0.05, **p < .01, ***p < .001

**Supplementary Material B**

*Effects of categorical interference and cumulative conceptual interference*

As it is known from the literature a systematic increase in the number of exemplars encoded in long-term visual memory results in a decrease in recognition performance (see Konkle et al., 2010; Mikhailova et al., 2021), in this section, we examine whether and to what extend this type of interference may trump effects arising from cumulative conceptual interference and if these two sources of interference interact with each other. Following Mikhailova, et al., 2021, we computed *Categorical Interference* as the frequency of exemplars, belonging to the same semantic category, seen up to the point of recognition, and then normalized it to range between 0 and 1 (i.e., dividing by the maximum), so making this variable perfectly comparable to Cumulative Conceptual Interference (please refer to the main text for the definition of this variable). We predict Hits as a function of Categorical Interference and Cumulative Conceptual Interference introduced as main effects and in interaction using a generalized linear-mixed effects model (binomial link). The random variables considered are Participant (71), Semantic Category (27) and Recognition Order (2) evaluated as intercepts and slopes. For the description of the model building and evaluation, please refer to either the previous section or the main text. We find significant main effects of both Categorical and Cumulative Interference, whereby their increase results in a systematic decrement of recognition performance (i.e., hits). However, we do not find any significant interaction between the two. This result confirms that indeed categorical interference is detrimental to recognition processes (e.g., Mikhailova, et al., 2021), but at the same time, cumulative interference contributes to it above and beyond it (refer to Table 2S for the results of the model). In practice, introducing categorical interference as a predictor of the hit rate did not trump the effect of cumulative interference (i.e., it did not explain the same variance in the data) and the two types of interference did not interact, which possibly implies that they independently mediate recognition processes.

To further assess that this finding holds, we performed yet another analysis where we first fit a generalized linear model predicting hits as a function of category interference, and then took the residuals of this model as the dependent measure for a linear-mixed effect model where we predict it as a function of cumulative interference using the same random structure selected before minus the slope terms associated with category interference. In this way, we make sure that any possible effect of category interference has already been accounted for. We fully corroborate the significant effect of cumulative interference on the residualised hit rates (β = -0.36, t-value = -2.99, p < 0.001), which confirms that its effect is independent of category interference.

**Table 2S**

Generalized Linear Mixed-effects Model for Hits (seen objects) as predicted by Cumulative Interference (a continuous variable from 0 to 1) and Category Interference (a continuous variable from 0 to 1). The random variables introduced as intercept and slopes were Participant (71), Semantic Category (27) and Recognition Order (1, 2).

| Dependent Variable | Predictor | β | Std. β | CI  (2.5%; 97.5%) | SE | z-value |
| --- | --- | --- | --- | --- | --- | --- |
| Hits | Intercept | 2.06 | 0 | 1.65; 2.47 | 0.16 | 9.93*** |
|  | Category interference | -4.51 | -2 | -5.73; -3.3 | 0.08 | -7.29*** |
|  | Cumulative interference | -0.39 | -0.23 | -0.66; -0.12 | 0.08 | -2.83** |

*Notes*: The final model formula in Wilkson notation, resulting from stepwise backward selection is:

‘Hits ~ Cumulative Interference + Category Interference + (1 | Participant) + (1 | Semantic Category) + (0 + Category Interference | Participant) + (0 + Category Interference | Semantic Category) + (0 + Category Interference | Recognition Order) + (0 + Cumulative Interference | Semantic Category)’

**Supplementary Material C**

*D-prime and criterion*

In this section, we conduct analyses of the d-prime and criterion to explore how the signal-to-noise ratio is influenced by the experimental variables of our design using the psych package in the R language (Revelle, 2023). We computed the d-prime and criterion for each participant according to our 2x2 factorial design. Statistical significance is obtained by modelling these two dependent measures as a function of Conceptual Relatedness (weak, strong) and Set Size (2, 4) using linear-mixed effect models where the random variable, introduced as intercept and slope is Participant (71). We followed the same procedure described throughout to build and evaluate our models. D-prime was significantly higher when target objects were encoded in sets of 4 compared to sets of 2. Criterion also was significantly higher for objects encoded in sets of 4, especially when the conceptual relatedness of the set was strong (refer to Table 3S for the model output and Figure 2S for a visualization). These results largely corroborate the main result of this study: interference may prove to have some upside in strengthening, rather than weakening, memory for object exemplars.

**Figure 2S**

Means and standard-deviation across participants for the D-prime (panel A) and Criterion (unseen objects, panel B) as a function of the Set Size (2, 4) arranged in the horizontal axis separately for the two levels of Conceptual Relatedness (*weak*, grey circle; *strong*, red triangle).

**
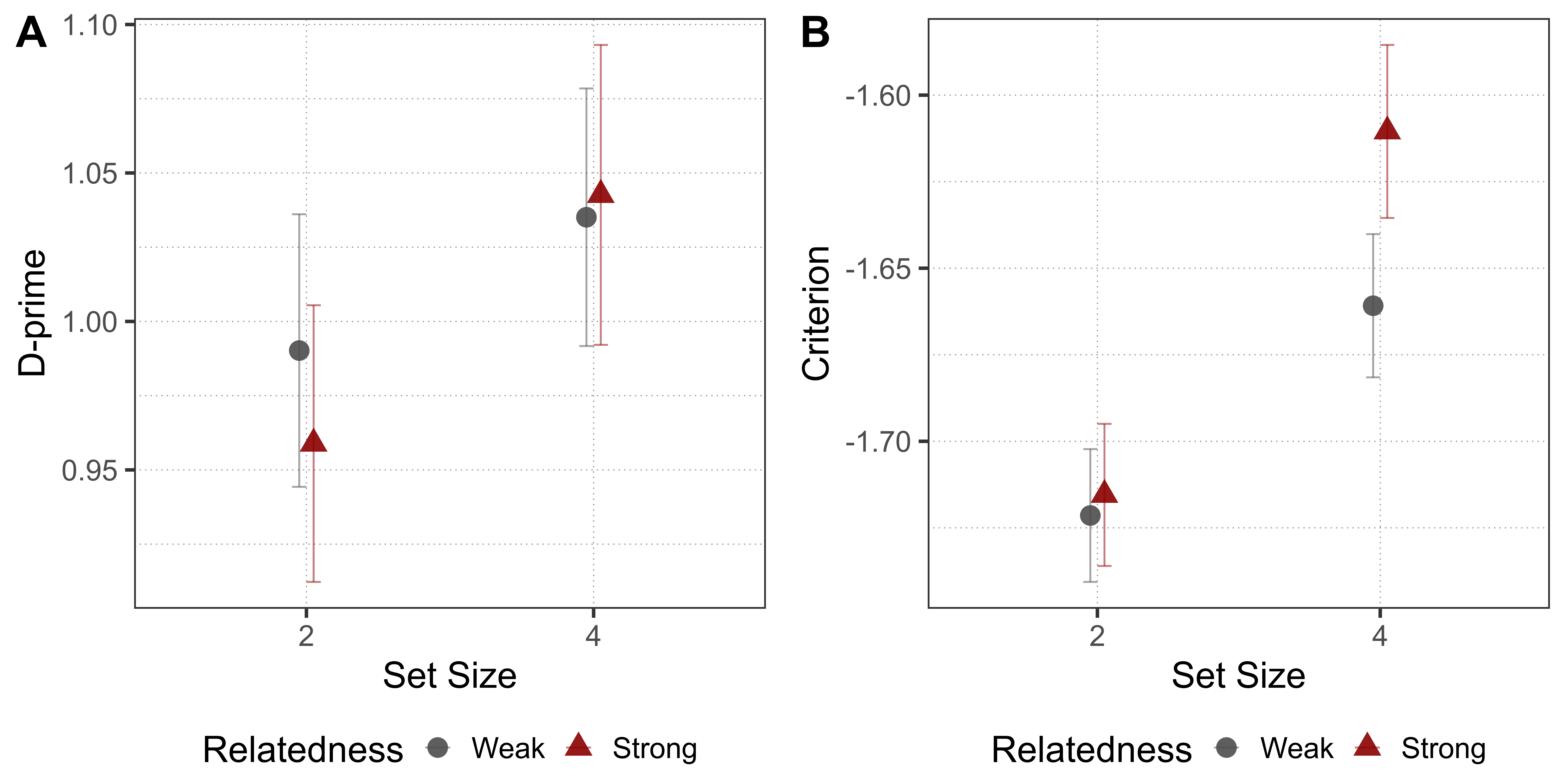
**

**Table 3S**

Generalized Linear Mixed-effects Model for D-Prime and Criterion as predicted by Set Size (2, 4, with 2 as the reference level) and Conceptual Relatedness (weak, strong, with weak as the reference level). The random variable introduced as intercept and slope was Participant (71).

| Dependent Variable | Predictor | β | Std. β | CI  (2.5% ; 97.5%) | SE | t-value |
| --- | --- | --- | --- | --- | --- | --- |
| D-prime | Intercept | 0.97 | 0 | 0.89; 1.05 | 0.04 | 23.37*** |
|  | Set Size (4) | 0.06 | 0.08 | 0; 012 | 0.03 | 2.18* |
| Criterion | Intercept | -1.72 | 0 | 0.28; 0.9 | 0.02 | -84.85*** |
|  | Conceptual Relatedness (strong) | 0 | 0.01 | 0.04; 0.44 | 0.01 | 0.43 |
|  | Set Size (4) | 0.06 | 0.16 | 0.17; 0.57 | 0.01 | 3.95*** |
|  | Set Size (4) x Conceptual Relatedness (strong) | 0.04 | 0.1 | 0.21; 0.58 | 0.02 | 2.27* |

*Notes*: The final model formulas in Wilkson notation, resulting from stepwise backward selection are:

a) D-prime ~ Set Size + (1 | Participant)

b) Criterion ~ Conceptual Relatedness + Set Size + Conceptual Relatedness x Set Size + (0 + Set Size | Participant)

(*) p < .10, *p < 0.05, **p < .01, ***p < .001

**Supplementary Material D**

*An alternative way to compute cumulative conceptual interference*

We computed cumulative interference as the sum of conceptual relatedness values between consecutive objects. This measure was then normalized to range between 0 and 1, dividing it by the maximum observed across the set. As we tested recognition of the last object of each set, an alternative way would be to sum instead the relatedness scores between each object and the last one only. So, we calculated cumulative conceptual interference in this way (again normalizing to range between 0 and 1) and used it to predict the hit rate in a generalized-linear mixed model with Participant (71), Semantic Category (27) and Recognition Order (2) evaluated as intercepts and slopes. We built and evaluated models following the same procedure amply reported above and in the manuscript. Our results fully replicate the analysis presented in the manuscript where cumulative conceptual interference is instead computed sequentially between objects of the same set (refer to Table 4S).

**Table 4S**

Generalized Linear Mixed-effects Model for Hits (seen objects) as predicted by Cumulative Interference (a continuous variable from 0 to 1) in its alternative formulation. The random variables introduced as intercept and slopes were Participant (71), Semantic Category (27) and Recognition Order (1, 2).

| Dependent Variable | Predictor | β | Std. β | CI  (2.5%; 97.5%) | SE | z-value |
| --- | --- | --- | --- | --- | --- | --- |
| Hits | Intercept | 0.78 | 0 | 0.46; 1.10 | 0.16 | 4.82 |
|  | Cumulative Interference | -0.65 | -0.21 | -0.65; -0.13 | 0.13 | -3.01** |

*Notes*: The final model formula in Wilkson notation, resulting from stepwise backward selection is:

‘Hits ~ Cumulative Interference + (1 | Participant) + (1 | Semantic Category) + (1 | Recognition Order) + (0 + Cumulative Interference | Semantic Category)’

**References**

Barr, D. J., Levy, R., Scheepers, C., & Tily, H. J. (2013). Random effects structure for confirmatory hypothesis testing: Keep it maximal. Journal of Memory and Language, 68(3), 255–278. https://doi.org/10.1016/j.jml.2012.11.001

Konkle, T., Brady, T. F., Alvarez, G. A., & Oliva, A. (2010). Scene memory is more detailed than you think: the role of categories in visual long-term memory. *Psychological Science*, *21*(11), 1551–1556. https://doi.org/10.1177/0956797610385359

Kuznetsova, A., Brockhoff, P. B., & Christensen, R. H. B. (2017). lmerTest Package: Tests in Linear Mixed Effects Models. *Journal of Statistical Software*, *82*(13), 1–26. https://doi.org/10.18637/jss.v082.i13

Matuschek, H., Kliegl, R., Vasishth, S., Baayen, H., & Bates, D. (2017). Balancing Type I error and power in linear mixed models. *Journal of Memory and Language*, *94*, 305–315. https://doi.org/10.1016/j.jml.2017.01.001

Mikhailova, A., Raposo, A., Della Sala, S., & Coco, M. I. (2021). Eye movements reveal semantic interference effects during the encoding of naturalistic scenes in long-term memory. *Psychonomic Bulletin & Review*, 1–14. https://doi.org/10.3758/s13423-021-01920-1

Revelle, W. (2023). *psych: Procedures for Psychological, Psychometric, and Personality Research* (R package version 2.3.9).

Searle, S. R., Speed, F. M., & Milliken, G. A. (1980). Population marginal means in the linear model: An alternative to least squares means. *American Statistician*, *34*(4), 216–221. https://doi.org/10.1080/00031305.1980.10483031
